# A Cross-Sectional Assessment of Effects of Imprisonment Period on the Oral Health Status of Inmates in Ghaziabad, Delhi National Capital Region, India

**DOI:** 10.7759/cureus.27511

**Published:** 2022-07-31

**Authors:** Puneet Kumar, Prince Kumar, Anushree Tiwari, Mimansha Patel, Soumeen Niteen Gadkari, Divya Sao, Kapil Paiwal

**Affiliations:** 1 Department of Public Health Dentistry, Shree Bankey Bihari Dental College & Research Centre, Ghaziabad, IND; 2 Department of Prosthodontics, Rama Dental College, Hospital, and Research Centre, Kanpur, IND; 3 Research Analysis, American Academy of Orthopaedic Surgeons, Rosemont, USA; 4 Department of Oral Pathology and Microbiology, Triveni Institute of Dental Sciences, Hospital and Research Centre, Bilaspur, IND; 5 Department Of Oral and Maxillofacial Surgery, Karmaveer Bhausaheb Hiray Dental College and Hospital, Nashik, IND; 6 Department of Oral Pathology, New Horizon Dental College & Research Institute, Bilaspur, IND; 7 Department of Oral Pathology and Microbiology, Daswani Dental College & Research Center, Kota, IND

**Keywords:** prison inmates, prison health, inmates, oral health, needs assessment, dental caries, dmf index

## Abstract

Background: Incarcerated individuals usually exhibit high oral health needs than the average population. Several factors contribute to these needs both before incarceration and during the sentence itself. Inmates are a marginalized group, who are at a higher risk for a variety of medical, dental, and emotional disorders than the general population. The aim of the study was to assess of effects of the imprisonment period on the oral health status of inmates.

Material and Methods: A total of 532 inmates with imprisonment up to three years, three to six years, and six to ten years were included in the study. Incidence and prevalence of dental caries, decayed, missing, filled teeth (DMFT) index, and periodontal and prosthetic status were evaluated in detail.

Results: Results showed that the prevalence of dental caries was relatively high among the convicts. It was found that 98.2% of the inmates had one or more teeth decayed. Additionally, 31.2% (pocket >4mm) of the inmates had poor periodontal status with 4.5% of the subjects having a loss of attachment score of 4-5mm or more. A total of 3.5% of the inmates had full dentures, either upper or lower arch. The relative need for full prosthesis was projected to be around 1.4% of the studied population.

Conclusion: Within the limitations of the study, the authors found that dental healthcare delivered and received by the inmates is much below the acceptable limit. Additionally, the incidence of dental caries in inmates was unexpectedly higher with tooth decay in 98.2% of subjects. Hence, the need of the hour is to critically incorporate and reinforce our efforts with a special focus on the risk factors of oral health.

## Introduction

Prison inmates are individuals subjected to imprisonment mostly for the purpose of security and to keep the peace or good behavior. A person is usually in custody for criminal charges and on trial. Inmates generally face severe social negligence along with unacceptability [1]. Moreover, most incarcerated individuals belong to low socioeconomic backgrounds with a history of insecurity and recent job loss. Their health status reflects this disadvantage [2]. Inmates bear a much higher burden of disease than other healthy people. They are at higher risk of various medical, dental, and emotional disorders than those in the general population [3]. They usually come across all levels of psychological, social, moral, and economic breakdown. This actually tends to enhance their negligence and carelessness towards general as well as oral health. Moreover, the relative risk of physical violence among themselves further deteriorates their physical and mental health to an irreversible stage [4]. The high need for oral health in incarcerated individuals may be attributed to several factors both before incarceration and during the sentence itself [5]. Many inmates are unemployed before being sentenced and come from communities with a high level of social exclusion [5]. Their higher needs and the nature of prison stays lead to increased levels of demand for emergency, urgent, and routine care. Prior to incarceration, most inmates have lower attendance at dental services than the general population. When they enter prison, the majority of the inmates have poor oral health [5]. All these may be due to low literacy levels and poor knowledge of oral health. They also have limited information about gum health and associated tooth disease [6]. Excessive alcoholism coupled with tobacco can enhance the incidence and seriousness of oral cancer [7].

Inmates are a vulnerable population comprising young people, elderly people, and people with physical disabilities. Internationally, a growing and changing prison population has opened up new avenues for research into inmates' oral health needs. Many of the researchers confirmed that the oral health requirements are truly imported by inmates and it is not the outcome of their life of imprisonment [2]. It is obvious that there are currently immense gaps in the literature on both the direct health effects and unintended consequences of imprisonment [7-10]. Therefore, the present study sought to assess the effects of the imprisonment period on the oral health status of inmates of a prison in the Delhi National Capital Region with simultaneous evaluation of relative dental caries experience, periodontal status, prosthetic status, and treatment needs of inmates.

## Materials and methods

The study was conducted in District Jail (Dasna), Ghaziabad, in the Delhi National Capital Region, India, to assess the effects of the imprisonment period on the oral health status of inmates using purposive sampling and a cross-sectional methodology [8]. The study was approved by Shree Bankey Bihari Dental College & Research Centre, Ghaziabad, India (approval number 07/IEC/DCPD/MDS/11-12/87). All the inmates present in the prison were screened and selected in the in-house dental unit of the District Jail (Dasna), Ghaziabad. Prior permission to conduct this study was obtained from the Superintendent, District Jail (Dasna), Ghaziabad. Voluntary written informed consent was also taken from all participants. Data were collected and recorded on a modified WHO 1997 proforma [9]. The tested and recorded parameters were: period of imprisonment, frequency of dental visits, past dental care received, dental care received during imprisonment, community periodontal status (CPI Index), dental caries status (DMFT (decayed, missing, filled tooth) Index), prosthetic status, and treatment need. Both genders were included in the study (male = 492, female = 40). Only those who were sentenced (inmates) were included in the study group.

Prior to the execution of data collection, the authors standardized the factors as per WHO criteria for identifying oral diseases. A group of subjects was then selected and examined for the full range of conditions expected to be assessed in the survey. Subjects were re-examined on successive days using the same diagnostic criteria. The results obtained were later subjected to the Kappa variability test. The mean Kappa co-efficient value for intra-examiner reliability with respect to Kappa co-efficient of all the indices used in the WHO Oral Health Assessment format was 0.88. The authors attempted the imperative clinical examination in which every participant was examined on a chair and the information was entered into a spreadsheet immediately [11]. All the diagnostic instruments were sterilized using an autoclave in the medical hospital of the prison. The investigator used disposable mouth masks and gloves during the examination as self-protection aids. The raw data were tabulated in a Microsoft Office Excel spreadsheet (Microsoft Corporation, Redmond, Washington, United States) before being entered into software for various statistical analyses, including descriptive statistics. Results of the study are prepared by estimating the mean, standard deviation, minimum and maximum, while other results are obtained in percentages (%). Significance levels are estimated at a 5% level of significance. A Chi-Square was utilized to calculate the importance of study factors amongst two groups.

All gathered data and details were entered in relevant tables of spreadsheets. The data sheets were sent for statistical analysis by IBM SPSS Statistics for Windows, Version 22.0 (Released 2013; IBM Corp., Armonk, New York, United States). The data was accurately processed by appropriate statistical tests. Different statistical parameters were calculated including p-values, mean, standard deviation, and chi-square test.

## Results

A total of 532 inmates were grouped according to the duration of their imprisonment: up to three years, three to six years, and 6-10 years. However, a major part of the study sample comprised incarcerated people imprisoned for only up to three years. Out of 532 inmates, 229 (43%) were in prison for up to three years, while 158 (29.7%) were in for three to six years, and 145 (27.3%) were imprisoned for 6-10 years. A total of 40 (7.5%) females and 492 (92.5%) males were examined and it was found that 340 (63.9%) of the inmates had not experienced dental operatory in their whole life. However, 104 (19.6%) of them had made past dental visits for dental treatments and their problems. Only a small proportion of inmates (n=114, 21.5%) experienced dental therapy in prison (Table 1).

**Table 1:** Distribution of study population based on various parameters of dental care

Frequency of dental visits	Number of subjects (n)	Percentage ( % )
Never	340	63.9%
Only if problem	192	36.1%
Distribution of study population based on past dental care received		
Past dental care received: Yes	104	19.6%
Past dental care received: No	428	80.4%
Distribution of study population based on dental care received during imprisonment		
Dental care received during imprisonment: Yes	114	21.5%
Dental care received during imprisonment: No	418	78.5%

In terms of the periodontal status, 168 (73.4%) of inmates imprisoned for up to three years had calculus, 38 (16.6%) shallow pockets (<5 mm), 11 (4.8%) deep pockets (>5 mm), and eight (3.5%) bleeding on probing. However, amongst the inmates imprisoned for three to six years only eight (5.1%) subjects had deep pockets with the majority having calculus (n=99, 62.7%) and shallow pockets (n=50, 31.6%). Of inmates imprisoned for 6-10 years, 84 (57.9%) showed clear evidence of calculus followed by 54 (37.2%), five (3.4%), and two (1.4%) subjects exhibiting shallow pockets, deep pockets, and bleeding on probing, respectively. Extremely significant values of community periodontal index (CPI) scores (p <0.001) were noticed wherein calculus was amongst the earliest pathological sign followed by pocket formation (Table 2).

**Table 2:** Periodontal status (CPI) of the study population according to period of imprisonment CPI: community periodontal index

CPI score	Up to 3 years (n=229, 43.0%)	3-6 years (n=158, 29.0%)	6-10 years (n=145, 27.3%)	Total N (N=532)
Bleeding (code 1)	08 (3.5%)	01 (0.6%)	02 (1.4%)	11 (2.1%)
Calculus (code 2)	168 (73.4%)	99 (62.7%)	84 (57.9%)	351 (66.0%)
Pocket 4-5mm (code 3)	38 (16.6%)	50 (31.6%)	54 (37.2%)	142 (26.7%)
Pocket 6mm (code 4)	11 (4.8%)	08 (5.1%)	05 (3.4%)	24 (4.5%)
Excluded sextant (x)	04 (1.7%)	00 (0.0%)	00 (0.0%)	04 (0.8%)

Table 3 shows fundamental statistical explanation with the level of significance assessment.

**Table 3:** Fundamental statistical description with level of significance evaluation using Pearson's chi-square test (for periodontal status)

Imprisonment	Mean	Std. Deviation	Std. Error	95% CI	Pearson Chi-Square Value	df	Level of Significance (p-value)
Up to 3 years	2.15	0.572	0.264	1.96	2.385	2.0	0.3278
3-6 years	2.26	1.030	0.042	1.96	2.254	1.0	0.0634
6-10 years	2.43	1.235	0.588	1.96	2.122	1.0	0.0200*

Table 4 shows a comparison among the three study groups using one-way ANOVA. 

**Table 4:** Comparison among the three study groups using one-way ANOVA (for group I, II, III, periodontal status)

Variables	Degree of Freedom	Sum of Squares ∑	Mean Sum of Squares m ∑	F value	Level of Significance (p-value)
Between groups	3	1.758	1.024	2.871	0.001*
Within groups	11	3.034	0.570	0	
Cumulative	61.43	9.804	0	0	

In addition, out of 24 subjects, loss of attachment of 4-5mm was found among 10 (4.4%), eight (5.1%), and five (0.9%) inmates imprisoned for up to three years, three to six years and 6-10 years, respectively. The authors noticed no statistically noteworthy values for attachment-loss scores among the inmates sentenced for various timings (p=0.306). With respect to dental caries, a total of 96.5%, 97.5%, and 94.5% of the inmates imprisoned for up to three years, three to six years, and 6-10 years, respectively, were affected. In the inmates imprisoned for three years, the mean score for decayed teeth was 4.77 ± 2.93 while the mean number of missing teeth was 0.89 ± 1.71, the mean number of filled teeth was 0.74 ± 1.14, and the mean DMFT score was 6.34 ± 4.04. A highly significant difference was observed in mean DMFT scores between the convicts imprisoned for different periods with higher scores for those imprisoned for longer periods (p < 0.001) (Table 5).

**Table 5:** Prevalence of dental caries (DMFT) according to period of imprisonment DMFT: decayed, missing, filled tooth; HS: highly significant; NS: not significant

Dental caries	Up to 3 years (N=229) (Mean ± SD)	3-6 years (N=158) (Mean ± SD)	6-10 years (N=145) (Mean ± SD)	Total (N=532) (Mean ± SD)	p-value
Decayed tooth	4.77 ± 2.93	5.32 ± 2.54	5.78 ± 2.64	5.21 ± 2.77	=0.002 (HS)
Missing tooth	0.89 ± 1.71	1.53 ± 1.88	1.57 ± 2.08	1.26 ± 1.89	< 0.001 (HS)
Filled tooth	0.74 ± 1.14	0.76 ± 1.15	0.70 ± 1.10	0.73 ± 1.13	= 0.888 (NS)
DMFT	6.34 ± 4.04	7.60 ± 3.70	8.03 ± 3.78	7.18 ± 3.93	< 0.001 (HS)

In the present study, the total mean number of decayed teeth was 5.21, the total mean number of missing teeth was 1.26, and the total mean number of filled teeth was 0.73 (Table 5). The majority of the study population had no prosthesis, i.e., 439 (82.5%) in the maxillary arch and 471 (88.5%) in the mandibular arch. The number of subjects who possessed maxillary prosthesis was 52 (9.8%) partial dentures, 27 (5.1%) bridge, and 13 (2.4%) full removable dentures while the figure for mandibular prosthesis was 40 (7.5%), 15 (2.8%) and 69 (1.1%), respectively. The prosthetic need of the convicts in the maxillary arch revealed the numeral of 60 (11.3%) with one unit prosthesis requirement, 33 (6.2%) with multi-unit prosthesis requirement, seven (1.3%) with a mixture of several unit prostheses, and three (0.6%) needed full prosthesis. In the lower jaw, the prosthodontic requirement of the inmates was 65 (12.2%) for one unit prosthesis, 40 (7.5%) for multi-unit prosthesis, 12 (2.3%) for a combination of one and/or multi-unit prosthesis, and four (0.8%) for full coverage prosthesis. Table 6 shows a fundamental statistical explanation with the level of significance assessment.

**Table 6:** Fundamental statistical description with level of significance evaluation using Pearson's chi-square test (for prevalence of dental caries)

Period of imprisionment	Mean	Std. Deviation	Std. Error	Pearson Chi-Square Value	Level of Significance (p value)
Up to 3 years	2.03	0.242	0.294	2.648	0.746
3-6 years	2.43	1.747	0.023	2.044	0.0650
6-10 years	2.86	1.751	0.595	2.246	0.0100*

Table 7 shows a comparison among the three study groups using one-way ANOVA. 

**Table 7:** Comparison among the three study groups using one-way ANOVA (for group I, II, III, prevalence of dental caries)

Variables	Degree of Freedom	Sum of Squares ∑	Mean Sum of Squares m ∑	F value	Level of Significance (p value)
Between groups	3	1.948	1.309	2.039	0.001*
Within groups	13	3.534	0.478	0	
Cumulative	70.13	8.637	0	0	

## Discussion

A few of the prime issues faced by incarcerated individuals are declining health, particularly oral health, which includes poor general and oral health [12]. In the Indian scenario, inmates have limited opportunities to consult a dental professional who could actually cater to an emergency with immediate and specialized dental healthcare. In our study, 36.1% of the inmates had experienced dental therapy, of which 19.6% were satisfied with the treatment. These findings are consistent with the findings of Nobile et al. [10], who assessed 544 inmates in Italy and discovered that 39.1% had received dental treatment, and Bansal et al. [11], who assessed 152 subjects and discovered that 36.8% had visited a dentist. They were, however, lower than the results reported by Osborn [12] (Australia), who included 789 inmates and stated that 62% had visited a dentist, and a study done by Nugent et al. [13], who assessed 516 inmates in a Scottish survey at Edinburgh, Scotland, and found that 69.8% of inmates had visited a dentist. The mean DMFT score in our study was noticed to be increased with an increase in the duration of imprisonment and this was comparable with the outcomes of the studies of Jones et al [14] who included 316 inmates in a survey in England and the DMFT score was 14.9, Osborn et al. [12] (Australia) who reported a DMFT score of 3.4, a survey done by Salive and Carolla [15] in the United States that included 178 inmates in which the DMFT score was 10.5, and Nugent et al. [13] in Scotland, who reported a DMFT score of 7.11.

In the current study, the occurrence of dental decay was higher amongst inmates imprisoned for three to six years and 6-10 years compared to those with up to three years of imprisonment. This may be due to the fact that, with an increased period of imprisonment, the incipient lesions that may be present before imprisonment become frank cavitations. The mean missing teeth in this study was 1.26, whereas, in a study conducted by McGrath [16], it was found to be 0.06, which was not in accordance with our study results. The possible reason could be the symptoms associated with dental visits. These visits were primarily aimed at dental treatment, especially extractions. The mean number of restored teeth in the current study was 0.73. The possible rationale may be the improper utilization of the available dental aids in the prison and a lack of knowledge, along with motivating factors towards preventive and curative dental treatment. While assessing the periodontal status with the CPI, not a single participant had a fit sextant. McGrath [16] and Clare [17] included 64 subjects and also reported that not a single prisoner had a zero CPI in Hong Kong Special Administrative Region (SAR), China.

Clare [17] noted that among inmates who had been incarcerated continuously for three years, there was a significant reduction in dental cavities. Compared to men, women had a much higher demand for tooth removals and fillings. This suggests that men use dental care more frequently than women do. The fact that female inmates may feel uneasy about entering a hospital section in jail that is mostly run by male inmates may help to explain why female inmates use oral healthcare facilities less frequently; the investigator saw this unease during interviews. Further research should be done in this area so that the authorities can get a particular suggestion [16].

In addition, 94.7% of the studied population showed an attachment-loss score of 0 (0-3mm). Only 4.3% of the study inmates had a score of 4-5 mm for attachment loss. It was discovered that 17.5% and 11.5% of the inmates had appliances in the upper and lower jaws, respectively, which is similar to the findings of Mixson et al. in the United States, who included 191 inmates and found that 17.3% of the inmates had maxillary prostheses and 5.2% had lower jaw appliances [18]. Gingival diseases that progress and are associated to calculus deposits may be the cause of periodontal disease [19]. This was in contrast to the study conducted by Nugent et al. in a Scottish survey in Edinburgh, Scotland, where up to 93% of edentate inmates reported partial or full dentures [13]. However, in the present study, the numbers of inmates with partial or full dentures were small, which may be due to the small numbers of inmates having denture prostheses, unawareness among the study population, and lack of dental care due to limited treatment accessibility [20].

With regards to prosthetic requirements, over 19.4% of inmates needed appliances in the upper jaw and 22.7% of inmates needed appliances in the lower jaw, which was somewhat comparable to a paper published by Uma and Hiremath [19] in Karnataka, India, where the sample size was 1309 inmates and they reported it to be 18.6% and 23.4% for maxillary and mandibular arches, respectively. It was also observed that 11.3% and 12.2% of the inmates needed at least one tooth replacement in the mandibular and maxillary arches, respectively. When seen in incarcerated subjects, 6.2% of the upper jaw and 7.5% of the lower jaw required one or more tooth restorations [21]. Various international studies have shown that the oral health condition of incarcerated people is poorer when compared with the general population and can overshoot into an epidemic situation in jails [22]. The study has observed various inmates and then evaluated.

The limitations of the current study include the fact that the sample size was small and details from other jails in the region had not been taken into consideration. Future studies should consider increasing the sample size and collecting detailed periodontal status for proper oral care.

## Conclusions

According to the results of our study, the quality and quantity of dental healthcare being delivered and received by inmates are much below the acceptable limit. The inmates had a high occurrence of dental decay with varying degrees of periodontal status. The need of the hour is to assimilate and reinforce dental healthcare efforts. Furthermore, an attempt has been made to provide baseline data on the oral health status of inmates for future assessments and comparisons. The cross-sectional nature of the study limited the ability to establish the time order of the risk factor and complications and future studies should keep this in view.

## References

[1] Heidari E, Dickinson C, Wilson R, Fiske J (2007). Verifiable CPD paper: oral health of remand prisoners in HMP Brixton, London. Br Dent J.

[2] (2007). Review of research on the effects of imprisonment on the health of inmates and their families. Review Of Research On The Effects Of Imprisonment On The Health Of Inmates And Their Families.

[3] (2009). Women’s health in prison; Correcting gender inequity in prison health. United Nations office on drug and crime. Women’s Health in Prison: Correcting Gender Inequity in Prison Health.

[4] Berkman A (1995). Prison health: the breaking point. Am J Public Health.

[5] (2007). Health in prisons: A WHO guide to the essentials in prison health. Health in Prisons: A WHO guide to the Essentials in Prison Health.

[6] Sode MK, Fareed N, Shanthi M, Sudhir KM (2011). Oral health status and treatment needs amongst prison inmates of Nellore district in Andhra Pradesh. J Ind Assoc Pub Heal Dent.

[7] Bedi K (2002). It’s Always Possible: Transforming One Of The Largest Prisons In The World. Sterling publisher’s pvt ltd.

[8] Peter S (2009). Essentials of Preventive and Community Dentistry. https://www.aryamedipublishing.com/Preventive_Community_Dentistry.htm.

[9] (1997). Oral Health Surveys: Basic Methods. Oral Health Surveys: Basic Methods.

[10] Nobile CG, Fortunato L, Pavia M, Angelillo IF (2007). Oral health status of male prisoners in Italy. Int Dent J.

[11] Bansal V, Sogi GM, Veeresha KL (2010). Assessment of oral health status and treatment needs of elders associated with elders' homes of Ambala division, Haryana, India. Indian J Dent Res.

[12] Osborn M, Butler T, Barnard PD (2003). Oral health status of prison inmates--New South Wales, Australia. Aust Dent J.

[13] Jones CM, McCann M, Nugent Z (2002). Scottish Prisons’ Dental Health Survey. Scottish prisons’ dental health survey.

[14] Jones CM, Woods K, Neville J, Whittle JG (2005). Dental health of prisoners in the north west of England in 2000: literature review and dental health survey results. Community Dent Health.

[15] Salive ME, Carolla JM, Brewer TF (1989). Dental health of male inmates in a state prison system. J Public Health Dent.

[16] McGrath C (2002). Oral health behind bars: a study of oral disease and its impact on the life quality of an older prison population. Gerodontology.

[17] Clare JH (2002). Dental Health status, unmet needs, and utilization of services in a cohort of adult felons at admission and after three years’ incarceration. J Correct Health Care.

[18] Mixson JM, Eplee HC, Feil PH, Jones JJ, Rico M (1990). Oral health status of a federal prison population. J Public Health Dent.

[19] Hiremath VP, Rao N (2011). Assessing the oral health status and treatment needs of the male inmates in the central prison of Karnataka state: a descriptive study. J Ind Assoc Public Health Dent.

[20] Hiremath VP (2009). Oral health status and treatment needs of the inmates in Hindalga Central Prison, Belgavi-Karnataka. J Ind Assoc Public Health Dent.

[21] Uma SR, Hiremath SS (2011). Oral health care for inmates of central prison, Bangalore- an institutionalized approach. J Ind Assoc Public Health Dent.

[22] Reddy V, Kondareddy CV, Siddanna S, Manjunath M (2012). A survey on oral health status and treatment needs of life-imprisoned inmates in central jails of Karnataka, India. Int Dent J.

